# Prediction of hepatocellular carcinoma risk in patients with type-2 diabetes using supervised machine learning classification model

**DOI:** 10.1016/j.heliyon.2022.e10772

**Published:** 2022-09-29

**Authors:** Noor Atika Azit, Shahnorbanun Sahran, Voon Meng Leow, Manisekar Subramaniam, Suryati Mokhtar, Azmawati Mohammed Nawi

**Affiliations:** a Department of Community Health, Faculty of Medicine, Universiti Kebangsaan Malaysia, 56000 Cheras, Kuala Lumpur, Malaysia; b Kuala Muda District Health Office, Ministry of Health Malaysia, 08000 Sungai Petani, Kedah, Malaysia; c Centre for Artificial Intelligence Technology (CAIT), Faculty of Information Science and Technology, Universiti Kebangsaan Malaysia, 43600 Bangi, Selangor, Malaysia; d Advanced Medical and Dental Institute (AMDI), Universiti Sains Malaysia, 13200 Kepala Batas, Penang, Malaysia; e Hepato-Pancreato-Biliary Unit, Department of Surgery, Hospital Sultanah Bahiyah, 05460 Alor Setar, Kedah, Ministry of Health Malaysia; f Hepato-Pancreato-Biliary Unit, Department of Surgery, Hospital Selayang, 68100 Batu Caves, Selangor, Ministry of Health Malaysia

**Keywords:** Hepatocellular carcinoma, Diabetes, Risk prediction, Machine learning, Support vector machine

## Abstract

**Background:**

Hepatocellular carcinoma (HCC) among type-2 diabetes (T2D) patients is an increasing burden to diabetes management. This study aims to develop and select the best machine learning (ML) classification model for predicting HCC in T2D for HCC early detection.

**Methods:**

A case-control study was conducted utilising computerised medical records in two hepatobiliary centres. The predictors were chosen using multiple logistic regression. IBM SPSS Modeler® was used to assess the discriminative performance of support vector machine (SVM), logistic regression (LR), artificial neural network (ANN), chi-square automatic interaction detection (CHAID), and their ensembles.

**Results:**

Subjects (N = 424) were split into 60% training (n = 248) and 40% testing (n = 176) groups. The independent predictors identified were race, viral hepatitis, abdominal pain/discomfort, unintentional weight loss, statins, alcohol consumption, non-alcoholic fatty liver, platelet <150 ×10^3^/μL, alkaline phosphatase >129 IU/L, and alanine transaminase ≥25 IU/L. The performances of all models differed significantly (Cochran’s Q-test,p = 0.001) but not between the ensembled and SVM model (McNemar test, p = 0.687). SVM model was selected as the best model due to its simplicity, high accuracy (85.28%), and high AUC (0.914). A web-based application was developed using the best model’s algorithm for HCC prediction.

**Conclusions:**

If further validation studies confirm these results, the SVM model’s application potentially augments early HCC detection in T2D patients.

## Highlights

• Hepatocellular carcinoma (HCC) in type-2 diabetes (T2D) is increasing.
• A web-based application using a machine learning model has been developed.
• The model has 85.28% accuracy and high AUC (0.914) in HCC prediction.
• Potential risk stratification tool in T2D clinic to predict HCC.
• HCC prediction in T2D clinic may aid early detection and improve survival.

## 1 Introduction

Diabetes mellitus (DM) is a serious global health concern, affecting almost 500 million people worldwide, and the population of affected individuals is expected to grow [1]. It is one of the established risk factors for hepatocellular carcinoma (HCC), the most common type for primary liver cancer. HCC is one of the leading causes of cancer death worldwide, causing a significant burden of disability and loss of life years [2, 3]. An increasing trend of DM-related HCC has been demonstrated in epidemiological studies in the past three decades, especially in regions with a high incidence of diabetes and metabolic diseases [4]. Approximately 90% of DM are categorised as type 2 diabetes (T2D) and affected patients demonstrated a threefold increased risk of HCC compared to the normal population [5]. A previous epidemiological study noted the population attributable fraction of DM could be as high as 36.6 % of total HCC in the United States and 24.5% in the global population [6, 7]. Given the increased prevalence of DM and obesity, HCC incidence will continue to rise in the future [7].

However, lower survival rates have been reported in patients affected with HCC and DM. A large cohort study conducted among HCC patients in Taiwan found that DM patients with HCC had a significantly lower survival rate than non-DM patients [8]. Specifically, 1, 3, and 5-year survival rates in DM patients were 56.8%, 26.4%, and 12.7% compared to 61.6%, 32.8%, and 18.8%, respectively in non-DM patients [8]. Another study found that DM was related to poorer HCC prognosis with pooled hazard ratios of 1.46 (95% confidence interval [CI], 1.29; 1.66) for overall survival, and 1.57 (95% CI, 1.21; 2.05) for disease-free survival [9]. These low survival rates resulted in a long-term impact on national productivity.

One of the major causes of poor HCC survival is late detection. A study conducted in Malaysia found that 86.7% of HCC patients were diagnosed at a late stage, resulting in a poor median overall survival time of only 1.9 months [10]. However, the early detection of HCC remains a challenge. According to the World Health Organization, a cancer control program should be implemented in a defined population by systematically implementing evidence-based cancer prevention, early detection, diagnosis, treatment, and palliative care. Comprehensive cancer control aims to suit the general population’s needs and the high-risk subpopulation. While comprehensive screening and treatment access may effectively treat many cancers in high-income countries, in low- and middle-income countries, late presentation and limited treatment availability are responsible for nearly 70% of cancer deaths [11]. HCC surveillance is one of the initiatives for early detection of the disease. The American Association for the Study of Liver Disease (AASLD) recommended surveillance for HCC using ultrasonography with or without alpha-fetoprotein every 6 months in the high-risk individuals: only in cirrhosis and some non-cirrhosis hepatitis B carrier [12].

However, in T2D patients, there is no established standard for risk stratification for HCC in current practice [13]. Patients with T2D are hardly diagnosed at an early stage despite regular visits to a DM clinic for check-ups. Given the increasing burden on healthcare amid the COVID-19 pandemic, the management of chronic diseases may be adversely affected, especially in developing countries [14]. Therefore, a simple and reliable risk stratification tool may aid clinicians in the early detection of T2D patients at risk of developing HCC and offer the most appropriate management to them.

Previous attempts to develop risk-score models were primarily conducted in developed countries such as Taiwan, the United Kingdom, and Korea [15, 16, 17]. The challenges to adopting these models were noted because some of the parameters used in the model were unavailable in the DM primary care setting, especially in less-resourced countries. Moreover, different risk profiles might be generated due to the heterogeneity in the HCC attributable risks among the study population, such as DM prevalence, viral hepatitis, and alcohol consumption. Despite the increasing adoption of machine learning (ML) in the medical field, the use of ML in HCC screening remains limited. The ML models were built in the past to improve patient care quality and reduce medical costs [18]. Previously, Rau et al. [16] utilised an artificial neural network (ANN) in their predictive model in the T2D population, thus showing a promising model performance. However, this model did not utilise the biochemical parameters which are routinely monitored in primary care settings in DM clinics. Therefore, given the demand for HCC risk stratification in the DM population for early HCC detection at DM primary care clinics, this study was conducted to develop and select the best ML classification model for predicting HCC risk in T2D patients.

## 2 Materials and methods

### 2.1 Data source

This study utilised data from electronic medical records in Hospital Selayang (HS) and Hospital Sultanah Bahiyah (HSB), the hepatobiliary referral hospitals in West Peninsular Malaysia. These hospitals also run DM outpatient clinics as primary care. Both hospitals employed the Total Hospital Information System which provides access to multidisciplinary medical records, including clinical documentation, laboratory, radiology, and pharmacy information systems. The data were collected from 1 July 2020 until 31 December 2020, which included sociodemographic, clinical characteristics, and biochemical profiles. This study obtained ethical approval from the Malaysian Ministry of Health’s Medical Research and Ethics Committee (NMRR-18-3704-45037) and the National University of Malaysia Faculty of Medicine Ethics Committee (JEP-2019-356), including an exemption from the requirement for informed consent.

#### 2.1.1 Dependent variable

The operational definition for cases in this study is the diagnosis of HCC, coded as ICD-10- C22.0 in the electronic medical record system. The clinical classification coding in the medical record system was done by the certified coders from the respective hospitals according to the ICD-10 classification. The outcome variable will be cross-checked with the clinician’s note in the medical records by the researcher. The diagnosis of HCC was based on radiological findings from either computed tomography (CT) or magnetic resonance imaging (MRI) with or without histologically confirmed; which is according to the American Association for the Study of Liver Disease (AASLD) guideline [12]. The operational definition of control is T2D patients with no HCC diagnosis in the electronic medical record system.

#### 2.1.2 Independent variables

The sociodemographic variables are age, sex and race. The clinical characteristics are; clinical symptoms at diagnosis (weight loss, lethargy, loss of appetite, abdominal pain/discomfort, jaundice), duration of DM, underlying comorbidities (hypertension, obesity/overweight [BMI ≥23.0 kg/m^2^], viral hepatitis, nonalcoholic fatty liver [NAFLD], cirrhosis and portal hypertension), history of blood transfusion, family history of malignancies, DM medications at diagnosis (metformin, glibenclamide, gliclazide, insulin), statins, antivirals for viral hepatitis, traditional medication, alcohol consumption and smoking. The biochemical profiles were white blood cells, red blood cells, haemoglobin, platelets, mean platelet volume, glycated hemoglobin (HbA1c) level, albumin/globulin ratio, total bilirubin, alkaline phosphatase, and alanine transaminase and serum creatinine level. The operational definition of these variables is available in the supplementary material (S1).

### 2.2 Study design and study population

A 1:1 case-control study was performed. From 1^st^ January 2012 to 30^th^ June 2018, a total of 212 adult patients (age ≥18 years) newly diagnosed with HCC and a prior diagnosis of T2DM were chosen as cases from the database. Exclusion criteria were patients without DM treatment records or those with multiple cancer sites. Then, the chosen cases were paired with controls of the same age from DM outpatient clinic electronic medical records between 1^st^ January 2012 and 30^th^ June 2018. The inclusion criteria for the control group were persons with a known diagnosis of T2DM who visited outpatient clinics in the same year as the matched case. Patients diagnosed with cancer or without DM treatment records were excluded. The detailed methodology of this study was previously described elsewhere [19].

### 2.3 Development of supervised machine learning (ML) classification model

**Figure 1:**
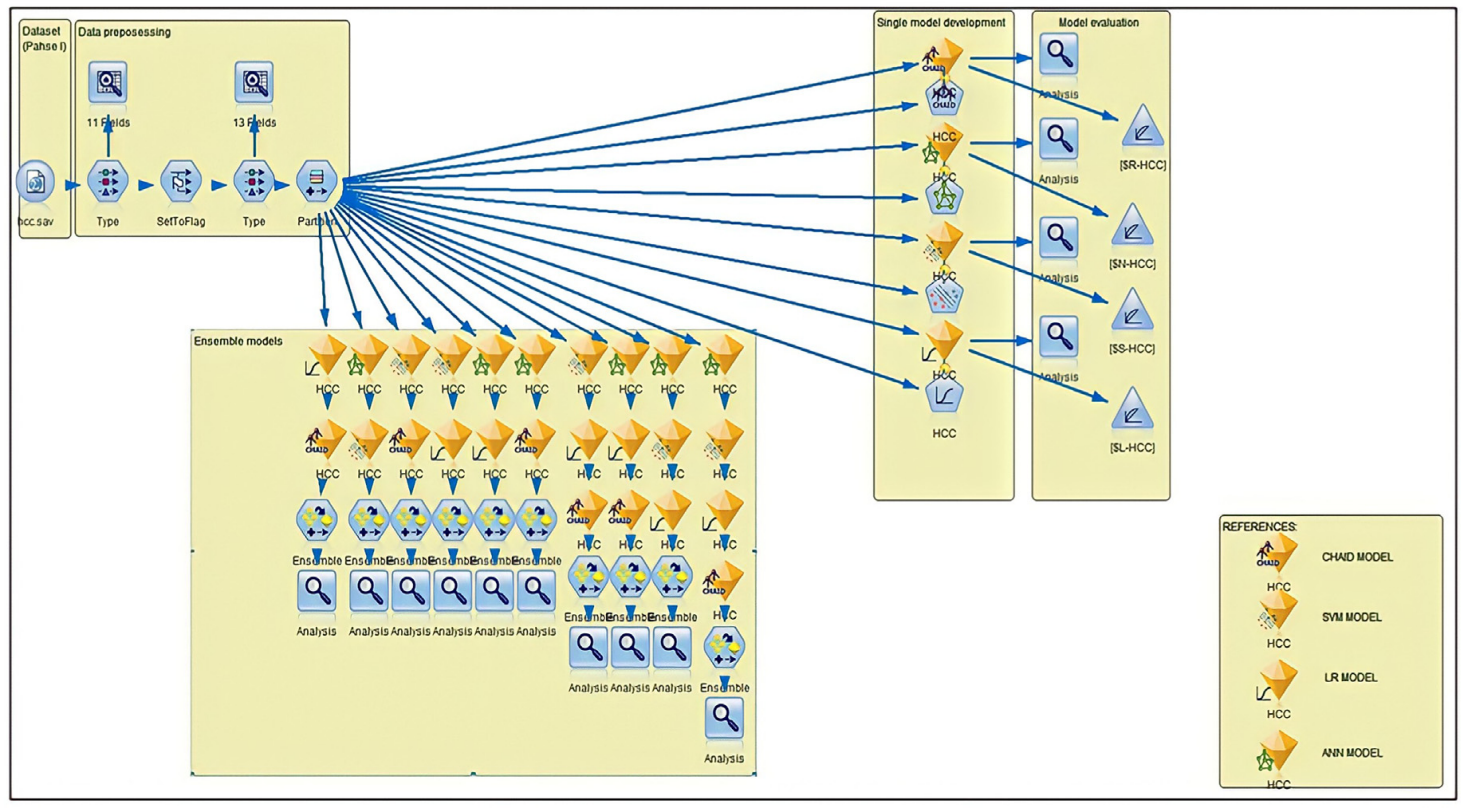
SPSS Modeler stream. Dataset file containing all the variables. In data processing, a type node was used to select the variables and to assign the appropriate categories. Data audit node was used to visualise the selected variables distribution and the validity of each variable. A SetToFlag node was selected for feature engineering, which involves converting nominal variables into categorical variables: “yes or no”. The transformed data were re-analysed using the data audit node.

The model development and comparisons were performed using the Statistical Package for Social Science (IBM® SPSS® Modeler version 18.0). The datasets collected were compiled and preprocessed, including variable selection, quality exploration, cleaning, and feature engineering. Next, the random partitioning was generated with 60% for model training, followed by 40% testing of the model. The model performance was evaluated and compared, before selecting the best model fit. Figure 1 shows the actual stream in the SPSS modeler user interface used in this study.

#### 2.3.1 The dataset

The multiple imputations technique were performed using IBM SPSS, version 21 for missing data. A fully conditional specification (FCS) method was employed to generate five imputed datasets. The missing data analysis was previously presented elsewhere [19]. The multiply imputed dataset was compressed into a single dataset using the “bar procedure”. This technical procedure was created by Baranzini [20], which facilitates the compression of several imputed data frame outputs into a single pooled data frame. This method has also been used by other researchers [21, 22].

#### 2.3.2 Data preprocessing

The data preprocessing include variable selection, data quality exploration, data cleaning, feature engineering, and partitioning the dataset.

(i) Variable selection

The association between independent variables (sociodemographic factors, biochemical profiles, and clinical characteristics) and HCC risk among T2D patients was evaluated using multivariate logistic regression analysis. A backward likelihood ratio method was used for the variable selection into the model, with probability for entry at 0.05 and removal at 0.10. The multicollinearity of the variables was checked using the variance inflation factor.

(ii) Data quality exploration

A data audit node in the software was used to assess the data quality and to describe the characteristics of the included variables. This process allowed the identification of any variables with missing values or invalid entries.

(iii) Data cleaning

Missing data management was unnecessary since the dataset was derived from previously imputed data [19]. All the values were legitimate for processing and there were no duplicates or outliers.

(iv) Feature engineering

For improved model performance, the nominal variable (race) containing three categories was transformed into three categorical (flag) variables using the “Set to Flag” node. The “flag” was the measurement set for true or false responses.

(v) Partitioning the dataset

The dataset was divided into a testing and training set using a random generator. The model capacity was verified by the testing set. This process resulted in generating new data by cross-validating the goodness of fit developed using the training dataset. A 50:50, 60:40, 70:30, 80:20, and 90:10 training: testing ratio was evaluated. The 60:40 training or testing ratio was selected for further analysis as the ratio created the most stable and high-performing model.

#### 2.3.3 Modelling

The four types of classification algorithms chosen for the model development were the support vector machine (SVM), logistic regression (LR), artificial neural network (ANN), and chi-square automatic interaction detection (CHAID). The combination of these models (ensemble model) was also evaluated. Table 1 represents the build setting of each developed model. The predictor importance was generated in the form of a graph for all the single classifiers to assess the relative importance of each predictor in model estimation.

**Table 1:** The build setting parameters for LR, ANN, SVM, CHAID, and ensembled models.

**Models**	**Parameters**
LR	Multinomial Method: Enter
	Singularity tolerance: 1.0E-8
	Maximum iterations:20
	Maximum step-halving:5
	Log-likelihood convergence:1.0E-1
	Parameter convergence:1.0E-6
	Delta:0.0
	Confidence interval: 95.0
ANN	Neural network model: Multilayer perceptron (MLP) Hidden Layers: Automatically compute the number of units Hidden layer 1: 1 Hidden layer 2: 0 Stopping Rules: Use maximum training time (per component model): true, Minutes: 15 (default) Advanced: Overfit prevention set (%): 30.0 (default) Missing values in predictors: Delete listwise Replicate Results: true Random seed: 365625423
SVM	Stopping criteria: 1.0E-3 Kernel type:RBF Regularization parameter: 2 RBF gamma: 0.1
CHAID	Levels below root:5 Alpha for Splitting:0.05 Alpha for Merging:0.05 Epsilon For Convergence: 0.001 Maximum iterations for convergence:100 Use Bonferroni adjustment: True Allow splitting of merged categories: No Chi-Square method: Pearson Stopping criteria: Use percentage Minimum records in parent branch (%): 2 Minimum records in child branch (%):1
ENSEMBLE	Ensemble method: Confidence-weighted voting Voting: random selection

#### 2.3.4 Model evaluation

(i) Model performances

After the model development, the confusion matrix and the receiver operating characteristic (ROC) curve were used to assess the model performance of the training and testing datasets [23]. Based on the confusion matrix, the number of true positives (TP), true negatives (TN), false positives (FP), false negatives (FN) were determined. The confusion matrix was used to calculate the accuracy, classification error, sensitivity, specificity, positive predictive value, and negative predictive value. On the other hand, the ROC curve plots the real positive rate versus the FN rate at various threshold points, whereas the discriminatory ability of the classification models was determined using the area under the ROC curve (AUC).

(ii) Model prediction stability

The model prediction stability was determined using the standard deviations of the accuracy of each classification model. The models were run 10 times with 10 different random seeds with the same settings and hyper-parameters as the previous counterparts. This is a typical process for reporting model performance across the community [24].

(iii) Significance test

A significance test was applied to assess the hypothesis for the classification model differences. Specifically, the differences between all models were determined using Cochran’s Q test. The McNemar test was then used to compare the two models with the best results [25].

#### 2.3.5 Development of web-based risk predictor for HCC in T2D

The best model was deployed using the IBM Watson Machine Learning application through the IBM cloud server. Next, an interactive web application (user interface) was created using Python version 3.10 and Streamlit 1.4.0. This application will receive and validate input from the user and send the data to the deployed model using IBM Application Programming Interface (API) for HCC prediction and display the predicted output to the user.

## 3 **Results**

### 3.1 Variables selection and characteristics

All 424 participants’ data were included in this study. The multiple logistic regression (MLR) analysis showed that ten variables were significantly associated with HCC development after adjustment for age, sex, race, DM duration, blood transfusion, smoking, traditional medication, metformin, gliclazide, insulin, HbA1c, RBC, WBC, total bilirubin, and creatinine. These independent factors are; weight loss (adjusted odd ratio [AOR] = 5.28, 95% CI: 2.29; 12.19), having abdominal pain/discomfort (AOR = 6.73, 95% CI: 3.34; 13.34), viral hepatitis infection which interacted with Malay (AOR = 11.77, 95% CI: 1.39; 99.79) and Chinese race (AOR = 37.94, 95% CI: 3.92; 367.61), non-alcoholic fatty liver disease (AOR = 3.29, 95% CI: 1.40; 7.76), statins usage (AOR = 0.37, 95% CI: 0.21; 0.65), history of alcohol consumption (AOR = 4.08, 95% CI: 1.81–9.22), reduced platelet level <150 × 103/μL (AOR = 4.03, 95% CI:1.90; 8.55), ALP level >129 IU/L (AOR = 2.17, 95% CI:1.17; 4.00) and raised ALT ≥25 IU/L (AOR = 2.11, 95% CI: 1.16; 3.86) [19]. The variance inflation factor (VIF) of all included variables ranges from 1.18-1.79, suggested no multicollinearity problem among the included variables [26]. This finding had been discussed in detail in the previous literature [19].

**Figure 2:**
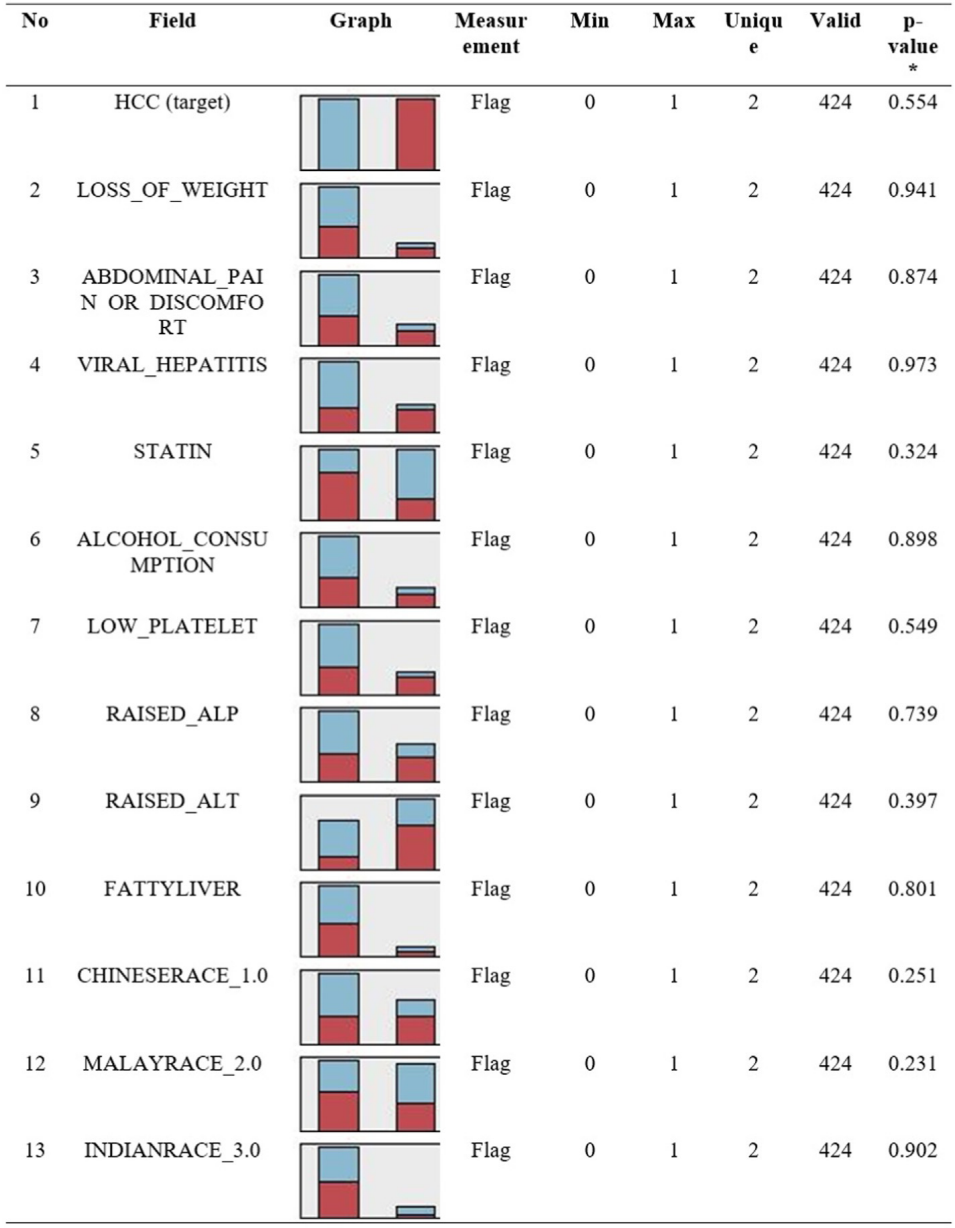
The characteristics of target and input variables included in the models. HCC status is the target variable, with the other 12 input variables. All were in the flag (yes/no) measurement. The graph colour in red indicates the proportion of variables with HCC = yes (1). No missing values for each variable. There was no significant different between training and testing set (p-value <0.05).

Based on the multiple logistic regression (MLR) model, the variables “race” and “viral hepatitis” were observed to interact in the final model. Nonetheless, these variables were included as two distinct variables in the ML algorithm to minimise the complexity of the final model to end-users (health practitioners). The input fields for the variable “race” were segregated into three categories: Chinese, Malay, and Indian. Figure 2 depicts the distribution of the included variables. The input fields for the variable “race” were segregated into three categories: Chinese, Malay, and Indian.

### 3.2 Logistic regression (LR) model

**Figure 3:**
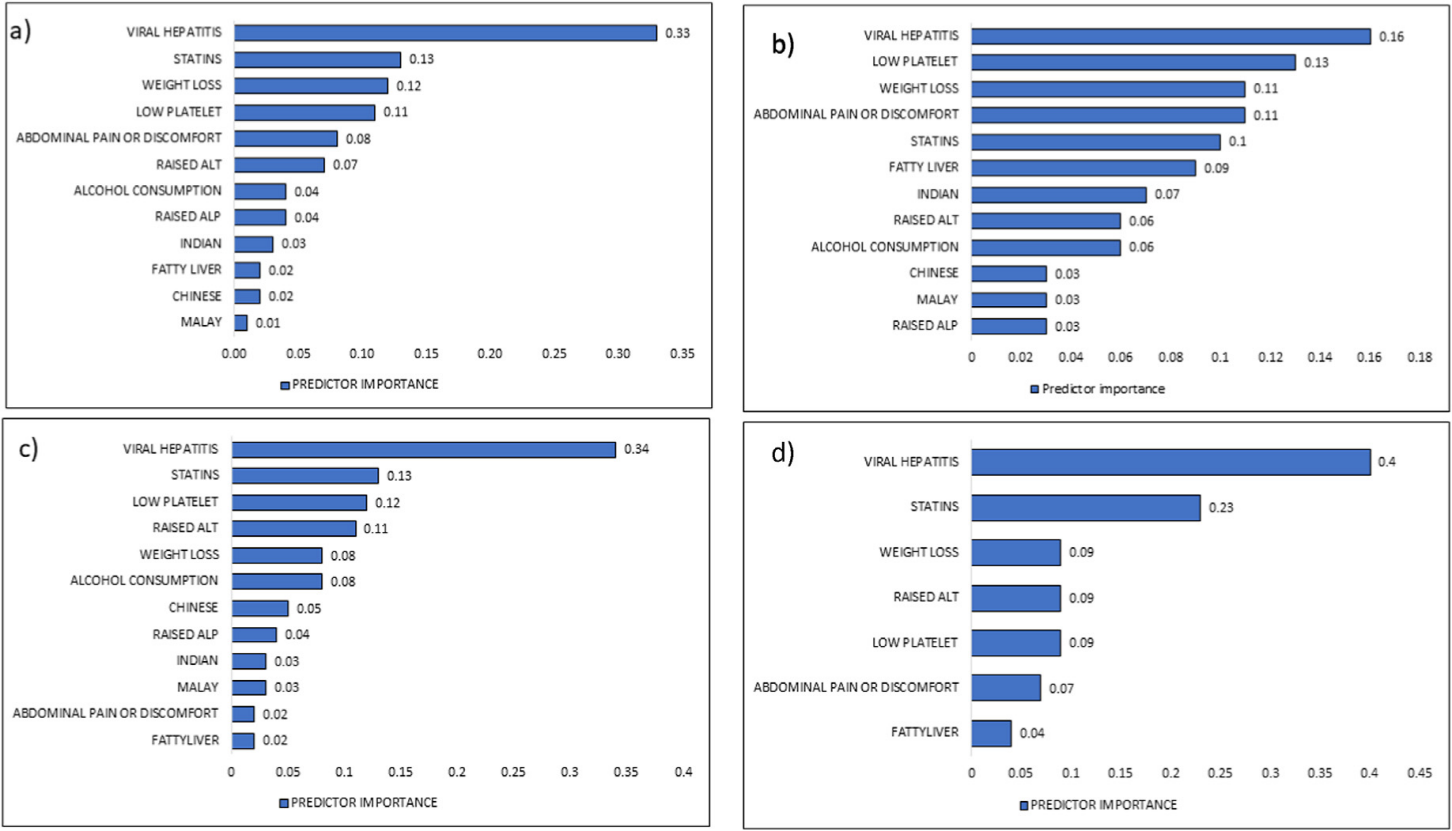
Predictor’s importance showing the relative contribution of each variable towards the model algorithm is presented as follows: a) LR-all input variables were included in the model with viral hepatitis contributing the most, b) ANN-viral hepatitis contributed the most to this model while ALP contributed the least c) SVM-all variables were included, with viral hepatitis contributing most to the models and d) CHAID models – only six variables were selected by the model out of 12 input variables in the final model, with viral hepatitis contributing the most.

The relative value of each predictor in estimating the LR model is depicted in Figure 3. (a). The presence of “viral hepatitis” is the most important predictor, followed by statins usage and weight loss. The equation derived by the logistic regression model to predict the outcome is available in Supplementary materials (S2).

### 3.3 Artificial neural network (ANN) model

As shown in Figure 3. (b), Viral hepatitis was the most important predictor of HCC diagnosis in the ANN model, followed by platelet level. The architecture of a multi-layer perceptron (MLP) neural network is shown in Supplementary materials (S3).

### 3.4 Support vector machine (SVM) model

Viral hepatitis was the most important predictor in the SVM model, followed by statins and platelet count. Figure 3. (c) shows the predictor importance calculated by the SVM algorithm.

### 3.5 Chi-square automatic interaction detection (CHAID) model

The CHAID model selected seven features. Figure 3. (d) shows that viral hepatitis was the most important predictor, followed by statins and weight loss. A decision tree diagram produced by the CHAID model in Supplementary materials (S4).

### 3.6 Ensembled models

Ensembled models were developed, and their performances were compared to that of single models. The top three ensembled models were combinations of four models (ANN, SVM, LR, and CHAID), three models (CHAID, LR, and SVM), and two models (ANN, and SVM) with high accuracy of 84.7%, 85.8%, and 85.5 %, respectively. The combination of CHAID, LR, and SVM models had the highest accuracy, with a testing set of 85.9% and a training set of 85.8%. In addition, the model also demonstrated an excellent AUC of 0.917; hence, it was used in the model comparison.

### 3.7 Comparison of the machine learning classification models’ performances

Table 2 provides a summary of the performance of the classification models and their ensembles. All models had more than 80% accuracy in their testing dataset, except for the CHAID model. The ensembled model had the highest accuracy (85.8%) in the testing set, followed by SVM (85.2%) and LR (84.7%). In terms of the stability depicted by the standard deviation, the SVM models were the most stable when it was run with ten different seeds generator. Excluding the CHAID model, all models had an AUC greater than 0.9 and the highest AUC (0.925) were recorded by LR. The LR and ensembled model had the highest sensitivity (83.5%), followed by the SVM and ANN models (82.4%) while the CHAID model had a relatively low sensitivity of 72.5%. SVM and ensembled models recorded the highest specificity (88.2%), whereas the LR model had the lowest (85.9%). Furthermore, the ensembled model had the highest PPV (88.4%), followed by the SVM model (88.2%) and the LR model (88.1%). Likewise, the ensembled model has the highest NPV (83.3%), followed by the LR model with 83.0% and the SVM model with 82.4%.

**Table 2:** Summary of the machine learning performance of the classification models.

Models	Dataset	N	TP	TN	FP	FN	Accuracy (%)	Standard deviation	C. error (%)	AUC	Sensitivity (%)	Specificity (%)	PPV	NPV
ENSEMBLED (LR, CHAID, SVM)	training	248	103	110	17	18	85.9	(±0.74)	14.1	0.919	85.1	86.6	85.8	85.9
	testing	176	76	75	10	15	85.8	(±1.19)	14.2	0.917	83.5	88.2	88.4	83.3
SVM	training	248	104	112	15	17	87.1	(±0.39)	12.9	0.926	86.0	88.2	87.4	86.8
	testing	176	75	75	10	16	85.2	(±0.78)	14.8	0.914	82.4	88.2	88.2	82.4
LR	training	248	101	108	19	20	84.3	(±1.03)	15.7	0.909	83.5	85.0	84.2	84.4
	testing	176	76	73	12	15	84.7	(±1.51)	15.3	0.925	83.5	85.9	86.4	83.0
ANN	training	248	100	108	19	21	83.9	(±0.81)	16.1	0.915	82.6	85.0	84.0	83.7
	testing	176	75	72	13	16	83.5	(±1.31)	16.5	0.905	82.4	84.7	85.2	81.8
CHAID	training	248	97	108	19	24	82.7	(±1.50)	17.3	0.879	80.2	85.0	83.6	81.8
	testing	176	66	72	13	25	78.4	(±1.96)	21.6	0.862	72.5	84.7	83.5	74.2

Abbreviations: SVM = support vector machine, LR = logistic regression, ANN = artificial neural network, CHAID = chi-square automatic interaction detection, TP = true positive, TN = true negative, FP = false positive, FN = false negative, C. error = classification error, AUC = area under the ROC curve, PPV = positive predictive value, NPV = negative predictive value.

### 3.8 Significance test

A statistically significant difference was observed between the models using Cochran’s Q test (Cochran’s Q = 23.91, df (4), p = 0.001). The McNemar test was applied to perform the pairwise comparison for the best two models (ensemble vs SVM). The p-value for this analysis was 0.687, indicating that the difference in performance between the ensembled and SVM model was not statistically significant.

### 3.9 Selection of the best model

Based on the result obtained, the SVM model was selected as the best model due to the following: (i) simplicity of the model (parsimonious) compared to the ensembled model (ii) had a stable model performance based on the standard deviation of its accuracy and no evidence of overfitting or underfitting (iii) a higher accuracy of 85.2% and higher discriminative ability (AUC = 0.914). In addition, the sensitivity of the model to detect HCC was 82.4% (true positive rate) while the specificity was 88.2%, indicating that it correctly rules out the HCC diagnosis when the prediction is negative.

### 3.10 Development of web-based risk predictor

**Figure 4:**
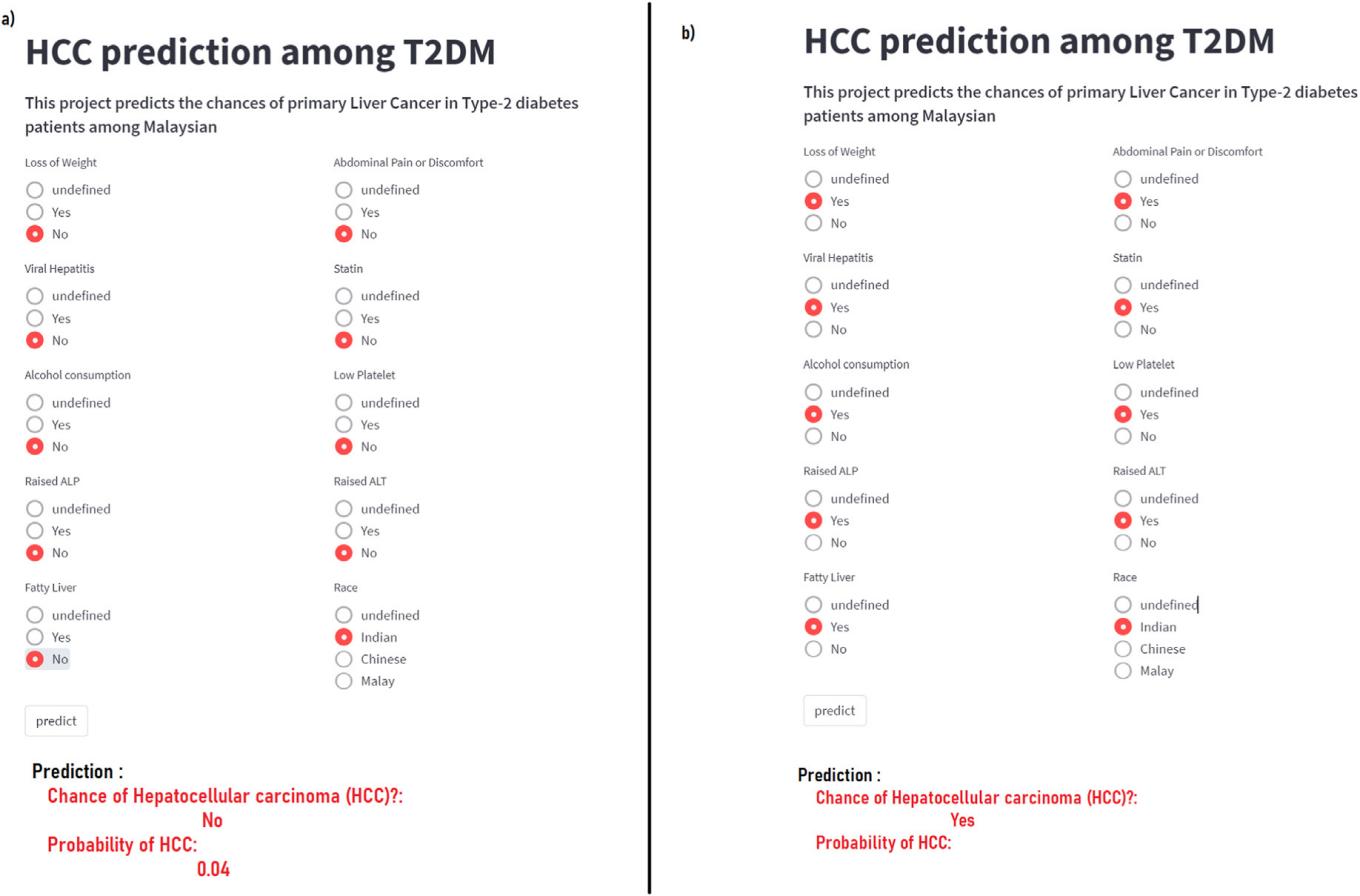
a) The web-based application with an example of the absence of any risk factors in an Indian patient. b) The HCC risk estimation in the presence of all the risk factors in an Indian patient.

A user-friendly web-based application was developed based on the SVM model’s algorithm (Figure 4.). This application is accessible at https://share.streamlit.io/predictor2021/hcc-predictor/main/main.py, consisting of ten radio multiple choice variables, which only allow a single answer for each variable. The application will provide the prediction of HCC and its probability once the input was submitted.

## 4 Discussion

This study aimed to develop and select the best supervised ML classification model to facilitate the early detection of HCC in T2D patients. The SVM model was selected as the best model due to its simplicity, stability and relatively efficient discriminative ability and performance. These characteristics enabled the model developed to be practically acceptable. It works by determining the best decision boundary for separating data points into groups and then using that boundary to forecast the class of future observations. The distinct groups may be separated by a linear straight line or a nonlinear border [27]. Among the ML models, the SVM appeared to represent a bridge between linear and nonlinear classification [28]. SVM is one of the most successful and adaptable classification algorithms available and capable of performing effectively in high-dimensional spaces [28, 29]. A previous study by Ko et al. found that viral hepatitis infection had synergistic interaction with DM in HCC development [30]. In the setting of a multiethnic Asian population, the current study noted multiplicative interaction between different races and viral hepatitis infection among the T2D population [19]. Hence, data patterns in HCC instances may exhibit complex interaction, necessitating a more flexible classification method to yield a more accurate classification prediction. Given the complexity of the HCC manifestation mechanism and the involvement of several carcinogenesis pathways and multiple risk factors in an individual, this SVM model is potentially useful in HCC prediction among T2D patients.

Nevertheless, since the SVM model exhibits the ML algorithm’s “black box” characteristic, it is usually difficult to comprehend the probability estimates of the included variables when the model is applied [23, 29]. This implies that the algorithm generated by the model is not straightforwardly interpretable to humans [31]. In contrast to the logistic regression model, where the odds ratio and coefficient may be used to explain the risk estimations of the variables towards the outcome. Thus, the predictor importance in the SVM model was computed to improve its interpretability. The predictor importance illustrates each included variable’s relative effect on predicting the outcome in the model estimation [32]. Viral hepatitis, the major risk factor for HCC [2, 12], was consistently on the highest importance list in all models. Statins were the second in the list except for the ANN model. In our study, statin was a protective factor for HCC, consistent with previous literature [33], and showed relative importance in HCC prediction among T2D patients.

As of 2020, at least five predictive models published in the literature forecasted HCC in the T2D population. The findings from these prior published studies are compared in Table 3. The model performance of the current study is consistent with other developed models. In terms of ML models, this study had an improved model performance compared to the ANN model developed previously. Nonetheless, all the reviewed models were developed for different target populations with varying input variables. This is useful in HCC risk stratification because different populations have distinct aetiological variances. [34].

**Table 3:** The comparison of the predictive models for HCC in T2D.

Authors	Country, race	Design	DM age-adjusted prev [1].	Viral hepatitis incidence [38]	Alcohol consumption [39]	Sample Size (N), Sample Pop.	Model	Variable(s)	Performance	Strength	Limitation
Current study 2021	Malaysia, Malay Indian Chinese	Case control	16.7%	1052.65/100 000 population	0.9 L/person	(N = 424) Case- 212 T2DM with newly diagnosed HCC Control- 212 T2DM without any cancer diagnosis	SVM, ANN, LR, CHAID	Race, symptoms (weight loss, abdominal pain/discomfort), viral hepatitis, statin, alcohol consumption, Alkaline phosphatase (ALP), Alanine transaminase, fatty liver disease	Best model, SVM ACC- 85.2 **AUC-0.914** SEN-82.4 SPEC-88.2 PPV-88.2 NPV-82.4	use ML classification models to evaluate the best predictors - unique multiethnicity variation -include sociodemographic, clinical and biochemical profiles available at primary care.	-retrospective study -Missing data
Grecian et al. 2020 [17]	Scotland, UK, Caucasian 98.3%	prospective cohort, 11 years follow up	3.9%	335.66/100 000 population	11.4 L/person	(N = 1059) T2DM 43 developed HCC/cirrhosis	the best prediction was the combination of USS screening and the fibrosis score Model performance analysed using LR	APRI AST, platelets >0.5.12 AST: ALT ratio FIB-4 age (years), AST, plt, ALT NFS age (years), BMI, IFG/diabetes, AST, ALT, platelet, albumin FLI triglycerides, BMI, γ GT, waist circumference	Best model: (APRI >0.5) AIC-291.6 C-STAT-0.82 SEN-80% SPEC-73% PPV-10% NPV-99% FP-27% FN-20%	-Large sample size, -prospective cohort	In a cohort with a moderately low cirrhosis/HCC existing risk scores did not reliably identify participants at high risk. -Complete case analysis was undertaken; Fibrosis is not the only pathway in HCC
Chen et al. 2019 [36]	China, Chinese	Case control	9.2%	3321.29/100 000 population	7.2 L/person	Model 1 (N = 200): Case: 79 T2DM with HCC Control: 121 T2DM patients without cancer Model 2(N = 259): Case: 79 T2DM with HCC Control: 180 T2DM with other cancers	LR , cross validation to evaluate performance	Gender, age, AST, direct bilirubin, GGT, triglyceride, total cholesterol, and hdl- cholesterol, uric acid,	**Model 1** Validation set **AUC-0.925** SEN -86.8%, SPEC-90.12%, ACC- 84.50% Model 2: Validation set AUC - 0.810 SEN-66.14%, SPEC-85.54%, ACC- 77.20%	Data coverage of 301 Hospitals	Missing data handling (impute with normal value) -not include hepatitis status, alcohol
Li et al. 2018 [15]	Taiwan, Chinese	retrospective cohort study	6.3%	N/A	N/A	(N = 31723) T2DM patients 748 HCC incident cases	cox -proportional hazard regression models	age, gender, smoking, variation in hemoglobin, serum glutamic–pyruvic transaminase, liver cirrhosis, hepatitis B, hepatitis C, antidiabetic medications, antihyperlipidemic medications, and total/high-density lipoprotein cholesterol ratio	Validation set: 3-year HCC risk **AUC -0.79**, 5-yea **AUC- 0.77** 10-year HCC AUC-0.76	-a large population-based study with a long-term follow-up period, -internal validation - risk score model	- missing data may be a potential bias - HBeAg, hepatitis B virus DNA or hepatitis C virus RNA levels (underestimate the strength of the predictor)
Si et al. 2016 [37]	Republic of Korea, Not mentioned	Retrospective cohort	6.3%	3832.50/100000	3.9 L/person	(N = 3544) DM without chronic viral hepatitis or alcoholic cirrhosis, 36 HCC incidences (N = 2364- derivation) (N = 1180- validation)	Cox proportional hazards model (DM-HCC risk score)	age >65 years, low triglyceride levels, and high GGT levels	Validation set 10-year development of HCC, **AUC- 0.86,** SEN 91.7%, SPEC 53.5%, PPV: 2%, NPV 99.8%	Involved large cohort of diabetic patients observed for a prolonged period of time. -utilised established electronic data warehouse -risk score model	Lacking of anti-HBc data in most of patients- high hepatitis B virus prevalence in Korea
Rau et al. 2016 [16]	Taiwan, Not mentioned	matched case-control	6.3%	4927/100000	N/A	2060 (case 515, control 1545) Newly diagnosed HCC with T2DM	ANN and LR	sex, age, alcoholic cirrhosis, nonalcoholic cirrhosis, alcoholic hepatitis, viral hepatitis, other types of chronic hepatitis, alcoholic fatty liver disease, other types of fatty liver disease, and hyperlipidemia	The performance of the ANN was superior to that of LR, **AUC-0.873** SEN-0.757 SPEC-0.755 SE - 0.014 PPV-0.730 NPV-0.790	web based application	did not use blood examinations as predictors

Besides the previous study conducted in China, the present study is among the first in developing countries. Due to an increased healthcare burden and the economic situation, cancer control in developing countries is faced with several challenges in terms of human resources, physical resources, and equipment [35]. A typical example is the limited availability of biochemical testing in primary care. Some laboratory tests were employed in previously constructed models, are not commonly performed in the primary care setting considered in this study, making it challenging to employ their approach. As a result, the medical information used in the present study as predictors included clinical symptoms, which have been found to contribute significantly to the model prediction. This represents the first attempt to include symptoms in the other five models. Although the symptoms are not specific and they may depict a late stage of the disease, their inclusion is vital for prompt patient management. Furthermore, this study has a unique variety of different races that may not apply to other countries; nonetheless, the application is still relevant in multiethnic Asian Pacific countries that bear a significant HCC burden [2].

The study strengths include being the first representative for a population with a high DM prevalence, intermediate hepatitis burden, and low alcohol use per capita in terms of the main attributable risk burden of HCC [1, 38, 39]. Except for the research conducted by Grecian et al. in the United Kingdom, most studies were carried out in countries with a high hepatitis B burden, moderate to high alcohol use, ranging from 3.9 to 11.9 L per person. In this study, viral hepatitis was still the main predictor for HCC in the T2D population; however, the effect varies significantly between different races. Therefore, the model developed in this study has a distinct target group compared to previously developed models.

**Figure 5:**
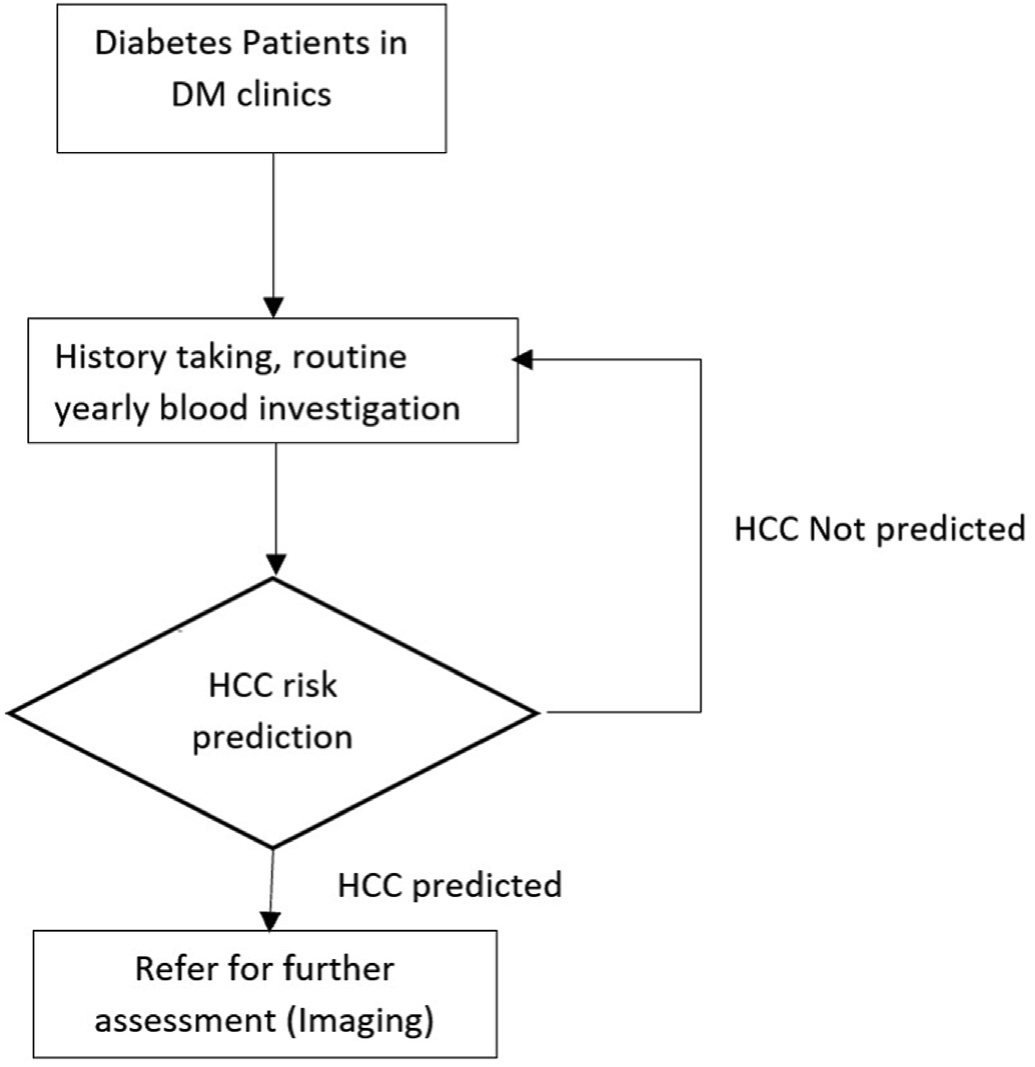
Patients in the T2D clinic who underwent routine check-ups and blood investigation will be assessed for HCC risk using the web-based HCC risk predictor. Patients who had been predicted for HCC need to be referred for further assessment including hepatobiliary imaging such as ultrasound. Those who had not been predicted will be assessed again in the next routine blood investigation.

This study used information that is widely available in clinical practice. The data was utilised to create an ML model that could classify patients at risk in the T2D population, with high accuracy and discriminative capacity. This methodology may help primary care physicians stratify high-risk patients for additional HCC surveillance. The web-based HCC predictor based on the best ML model in this study may provide a practical solution for HCC risk stratification in a busy T2D clinic in the future. Figure 5 shows the suggestion for T2D screening in the clinical setting. Thus, this model should be externally validated in a different population before the predictive model can be deployed in the clinical setting.

Besides, a study among the Asian population who live in the US noted that the maritime South East Asia population (comprised of Malaysian, Singaporean, Indonesian, and Filipino descent) had the highest cryptogenic HCC (15%), more likely to be symptomatic and has the lowest 10-year survival rates compared with other Asian’s subgroups [40], this study has potential to be tested in another geographical setting.

The study limitations are well-acknowledged. Firstly, the retrospective data gathering might result in missing data in several circumstances. To avoid extensive missing data, subjects with insufficient data were removed from the study onset to ensure the correctness of data analysis. In addition, a thorough multiple data imputation technique was applied to conserve cases with minimum missing data. This technique has improved data precision in previous studies [41, 42, 43]. Secondly, during the variable selection process, multiple explanatory variables were included to adjust the multivariate logistic regression model, increasing the risk of inflated standard errors of the model. However, the variance inflation factor was less than 2.5 for all the included variables, indicating a low risk for multicollinearity [26], thus reducing inflated errors of the model. Furthermore, to increase the model's interpretability, the statistical method was utilised for variable selection, whereas expert judgement is required to develop a practical model in the healthcare setting. Given the small sample size in relation to the number of variables in this work, we do not use full-automation of variable selection in ML [44]. Thirdly, even though cirrhosis is an important risk factor [45], it was not included in the final model because the variable was limited in the primary care setting and therefore difficult to be utilised. This is due to the fact that cirrhotic patients were referred to gastroenterologists/hepatologists and managed in the secondary care clinic, where designated HCC surveillance protocol was already established [13, 46]. Lastly, even though the inclusion of clinical symptoms increases the predictive accuracy of the current model, the nature of the information gathered from medical history is not specific and could be associated with the late stage of the disease. However, abdominal pain and weight loss were reported among the commonest symptoms presented at primary care by HCC patients in the previous literature [47]. Therefore, the variable was included as a more vigilant diagnostic work-up guide.

## 5 Conclusion

This study identified the SVM model with a high model performance value that was internally validated by utilising the medical data from the DM clinic. If externally validated, this model potentially could be employed as a personalised HCC risk stratification tool among T2D patients in primary care in the future while improving clinical judgment for early HCC diagnosis in this high-risk population.

### 5.1 Ethics

This study was carried out following the Helsinki Declaration and the Malaysian Good Clinical Practice Guideline. This study was approved by the Malaysian Ministry of Health’s Medical Research and Ethics Committee (NMRR-18-3704-45037) and the National University of Malaysia Faculty of Medicine Ethics Committee (JEP-2019-356), including an exemption from the requirement for informed consent.

## Declarations

### Author contribution statement

Noor Atika Azit: Conceived and designed the experiments; Performed the experiments; Analyzed and interpreted the data; Wrote the paper.

Shahnorbanun Sahran: Analyzed and interpreted the data; Wrote the paper.

Voon Meng Leow, Manisekar Subramaniam, Suryati Mokhtar: Performed the experiments.

Azmawati Mohammed Nawi: Conceived and designed the experiments; Analyzed and interpreted the data; Wrote the paper.

### Funding statement

Azmawati Mohammed Nawi was supported by 10.13039/501100004515Universiti Kebangsaan Malaysia [FF-2019-254].

### Data availability statement

Data included in article/supp. material/referenced in article.

### Declaration of interest’s statement

The authors declare no conflict of interest.

### Additional information

No additional information is available for this paper.
