# Supplementary material for: Prediction of hepatocellular carcinoma risk in patients with type-2 diabetes using supervised machine learning classification model
Source: Heliyon. 2022 Sep 29;8(10):e10772. doi: 10.1016/j.heliyon.2022.e10772 (PMC9529545; doi:10.1016/j.heliyon.2022.e10772)
Supplement: Supplementary materials [file mmc1.docx]

**Supplementary materials**

**Supplementary 1 (S1)**

**Table 1:**

**The operational definition of independent variables.**

| Variable | Operational Definition | Measures |
| --- | --- | --- |
| **Sociodemographic characteristics** | |  |
| Age | At the year of diagnosis (case) or year of enrolment (control) | Continuous (years) |
| Sex | Biological sex | Male; Female |
| Race | According to three main races in Malaysia | Malay; Chinese; Indian |
| **Clinical characteristics** | |  |
| Weight loss | Complaints of unintentional reduced weight irrespective of magnitude and duration documented in the medical records at diagnosis/enrolment | Yes; No |
| Loss of appetite | Complaints of reduced appetite documented in the medical records at diagnosis/enrolment | Yes; No |
| Abdominal pain/discomfort | Complaints of upper abdominal pain or discomfort documented in the medical records at diagnosis/enrolment | Yes; No |
| Jaundice | Complaints of yellowish discolouration of skin/jaundice documented in the medical records at diagnosis/enrolment | Yes; No |
| History of blood transfusion | Recorded of transfusion for blood or blood-related product in the medical records | Yes; No |
| History of alcohol consumption | Recorded of history of alcohol intake irrespective of the amount | Yes; No |
| Smoking | Documented smoking status as in medical record | Yes; No |
| Traditional medication | Recorded history of taking non-prescribed traditional medicine | Yes; No |
| Family history of malignancies | Recorded family history of any cancers | Yes; No |
| Non-alcoholic fatty liver disease (NAFLD) | The diagnosis of NAFLD diagnosed by clinician or evidence of fatty liver in the radiological report, with exclusion to excessive alcohol intake in the history. | Yes; No |
| Cirrhosis | The diagnosis of cirrhosis diagnosed by a clinician or in the radiological report. | Yes; No |
| Ascites | Clinically diagnosed ascites by physician documented in the medical notes or reported by the radiologist in the radiological reports. | Yes; No |
| Portal hypertension | The diagnosis of portal hypertension diagnosed by radiological findings. | Yes; No |
| Hypertension | Previous diagnosis of hypertension or on antihypertensive medication or BP>140/90 for two readings in the medical record. | Yes; No |
| Duration of DM | Number of years from diagnosis of diabetes to the diagnosis of HCC for cases, or number of years of diabetes to the enrolment year for controls. | More than 10 years; 0–9 years |
| Treatment of DM | Medication received for diabetes management at the time of presentation. | Metformin; gliclazide; glibenclamide; Insulin |
| Statins | Recorded any type of lipid-lowering drug under statins group at the diagnosis/ascertainment. | Yes; No |
| Antiviral (hepatitis treatment) | Recorded antiviral medication for viral hepatitis B/C | Yes; No |
| Viral Hepatitis | Diagnosis of viral hepatitis B/C based on the clinician’s diagnosis confirmed with blood investigation. | Yes; No |
| Body Mass Index ,BMI (kg/m^2^) | The value of weight divided by height square in metre (kg/m^2^). The categorisation of the BMI is according to the WHO recommendation of BMI classification for the Asian adult population [1]. | Normal: 18.5–22.9; Overweight/obese: ≥23.0 |
| **Biochemical profiles** | |  |
| White Blood Cell, WBC (×10^3^/µL) | White blood cell counts in a unit of ×10^3^/µL at the time of diagnosis. Cut-off point reference from Riley et al. [2]. | >11; ≤ 11 |
| Red Blood Cell, RBC (×10^6^/µL) | Value of red blood cells in a unit of ×10^6^/µL at the time of diagnosis. The categories were based on Xie et al. [3]. | Male: high: ≥4; low <4  Female: high: ≥3.5; low: <3.5 |
| Haemoglobin (g/dL) | Level of haemoglobin measured in g/dL at the time of diagnosis/enrolment. Categories reference was from Birgegard et al. [4]. | ≥12;<12 |
| Platelet (×10^3^/µL) | Level of platelet measured in ×10^3^/µL at the time of diagnosis/enrolment. Categories reference was from Chang et al. [5]. | <150; ≥150 |
| Mean Platelet Volume (fL) | Level of mean platelet volume measured in fL at the time of diagnosis. The categories were based on Scheiner et al. [6]. | >11; ≤11 |
| Glycated haemoglobin (HbA1c) level (%) | The HbA1c level at the last follow-up before HCC diagnosis (case) or enrolment (control). Cut-off point based on reference from Li et al. [7]. | ≥8.5;<8.5 |
| Albumin Globulin Ratio (AGR) | The ratio between the albumin and globulin in the liver function test at the time of diagnosis, calculate as serum albumin/ (total protein - albumin). Categories reference was from Suh et al. [8]. | <1.1; ≥1.1 |
| Total Bilirubin (µmol/L) | Level of total bilirubin in the liver function test at the time of diagnosis, measured in µmol/L. Categories reference was from Levick et al. [9]. | ≥21;<21 |
| Alkaline Phosphatase ALP (IU/L) | Level of Alkaline Phosphatase (liver enzyme) in the liver function test at the time of diagnosis/enrolment. The cut-off point of ALP is according to the local laboratory (normal range is 40-129). | >129; ≤129 |
| Alanine Transaminase, ALT (IU/L) | Level of alanine transaminase (liver enzyme) in the liver function test at the time of diagnosis/enrolment. The categories were based on literature from Hung et al. [10]. | ≥25; <25 |
| International normalised ratio, INR | The level on the international normalised ratio in the coagulation profile at the time of diagnosis. The categories were based on literature from Pagana et al. [11] | >1.2; ≤1.2 |
| Creatinine (µmol/L) | Creatinine level at the time of diagnosis/enrolment. Categories according to the local laboratory reference ranges. | Male: (low/normal/high) <5 /59–104/>104 Female : (low/normal/high)<45/ 45-84/ >84 |

References

[1] WHO Expert Consultation Appropriate body mass index for Asia populations and its implications for policy and interven-tion strategies. Lancet. 2004;363:157–63. DOI: 10.1016/S0140-6736(03)15268-3

[2] Riley, L. K. & Rupert, J. Evaluation of Patients with Leukocytosis Am Fam Physician 2015, 92(11): 1004–1011.

[3] Xie, X., Yao, M., Chen, X., Lu, W., Lv, Q., Wang, K., Zhang, L., et al. Reduced Red Blood Cell Count Predicts Poor Survival after Surgery in Patients with Primary Liver Cancer. Medicine 2015, 94(8): e577. DOI: 10.1097/MD.0000000000000577

[4] Birgegård, G., Aapro, M. S., Bokemeyer, C., Dicato, M., Drings, P., Hornedo, J.,Krzakowski, M., et al. Cancer-Related Anemia: Pathogenesis, Prevalence and Treatment. Oncology 2005, 68, 3–11.

[5] Chang, K. C., Hung, C. H., Lu, S. N., Wang, J. H., Lee, C. M., Chen, C. H., Yen, M. F., et al. A Novel Predictive Score for Hepatocellular Carcinoma Development in Patients with Chronic Hepatitis C after Sustained Response to Pegylated Inter-feron and Ribavirin Combination Therapy. J Antimicrob Chemother 2012, 2766–2772. DOI: 10.1093/jac/dks269.

[6] Scheiner, B., Kirstein, M., Popp, S., Hucke, F., Bota, S., Rohr-Udilova, N., Reiberger, T., et al.Association of Platelet Count and Mean Platelet Volume with Overall Survival in Patients with Cirrhosis and Unresectable Hepatocellular Carcinoma. Liver Cancer 2019, 8, 203–217. DOI: 10.1159/000489833.

[7] Li, T.-C., Li, C.-I., Liu, C.-S., Lin, W.-Y., Lin, C.-H., Yang, S.-Y. & Lin, C. Risk Score System for the Prediction of Hepatocellular Carcinoma in Patients with Type 2 Diabetes: Taiwan Diabetes Study. Semin Oncol, 2018, 45, 264-274. DOI: 10.1053/j.seminoncol.2018.07.006.

[8] Suh, B., Park, S., Shin, D. W., Yun, J. M., Keam, B., Yang, H. K., Ahn, E., et al. 2014. Low Albumin-to-Globulin Ratio Associated with Cancer Incidence and Mortality in Generally Healthy Adults. Ann Oncol 2014, 25, 2260–2266. DOI: 10.1093/annonc/mdu274

[9] Levick, C. How to Interpret Liver Function Tests. South Sudan Medical Journal 2017, 10, 40–43, doi:10.4314/ssmj.v10i2.

[10] Hung, Y.-C.; Lin, C.-L.; Liu, C.-J.; Hung, H.; Lin, S.-M.; Lee, S.-D.; Chen, P.-J.; Chuang, S.-C.; Yu, M.-W. Development of Risk Scoring System for Stratifying Population for Hepatocellular Carcinoma Screening. Hepatology 2015, 61, 1934–1944, doi:10.1002/HEP.27610.

[11] Pagana K.D.; Pagana T.J.; Pagana T.N. Mosby’s Diagnostic & Laboratory Test Reference. 14th Ed.; Elsevier: St. Louis, 2019.

**Supplementary 2 (S2)**


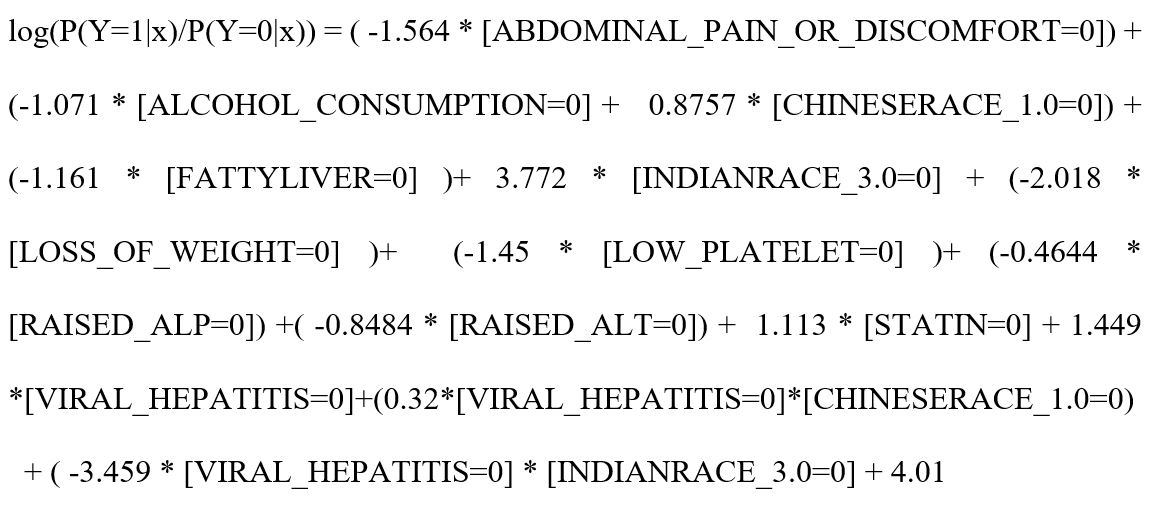


**Figure 1:** The Logistic regression equation model

--------------------------------------------------------------------------------------------------------------------------------------

**Supplementary 3 (S3)**


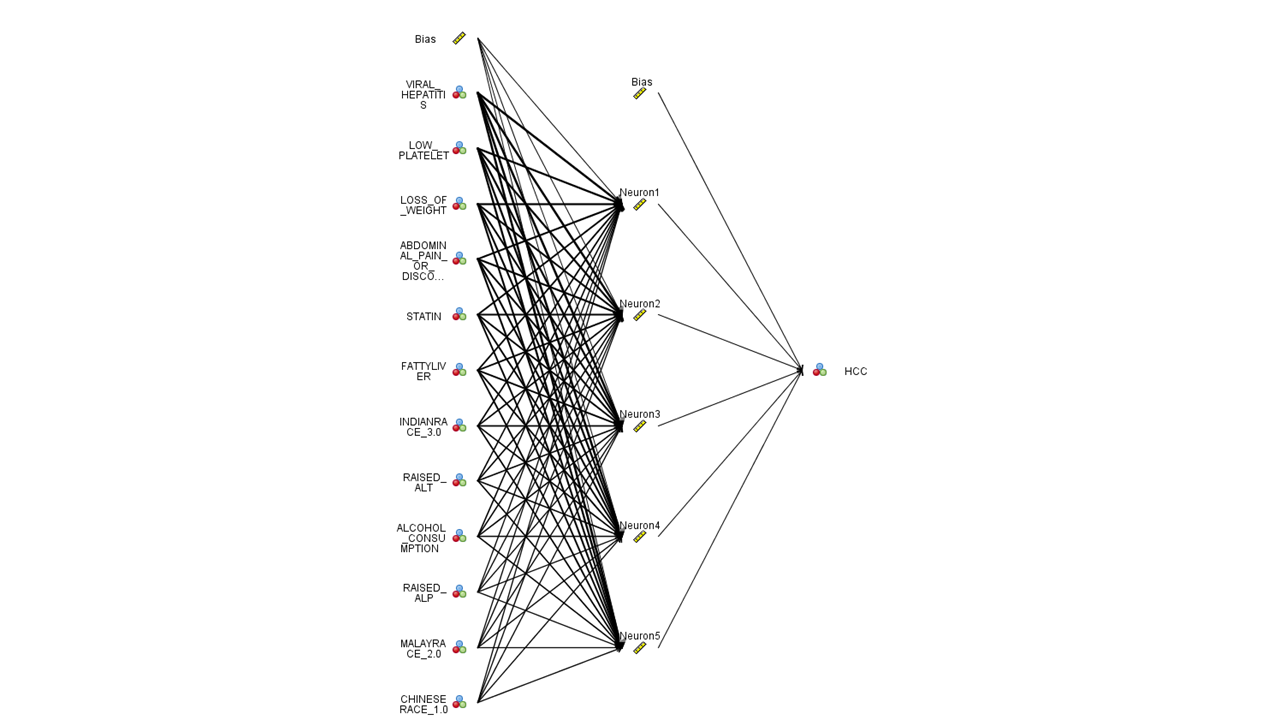


**Figure 2.** Architecture of the artificial neural network (ANN) model. A total of 12 variables were included in the study and the input layer was represented by one node for bias. The input layer transferred the weighted signals to the neurons in the hidden layer, leading to the formation of five neurons. The neurons were used as activation functions to convert the sum of the weighted signals into new information (output layer), thereby classifying the HCC status.

**Supplementary 4 (S4)**


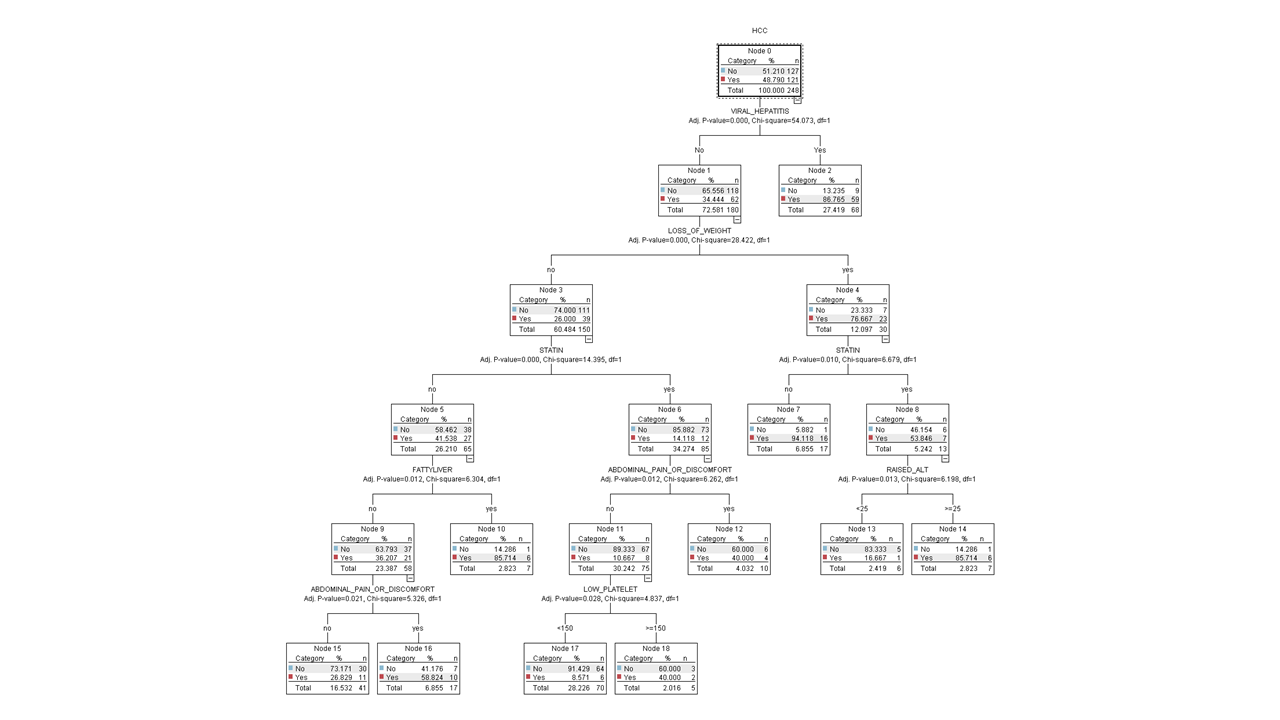


**Figure 3.** The decision tree diagram with the tree’s root node depicts the HCC status for the Chi-square Automated Interaction Detection (CHAID) model. The most important predictor (viral hepatitis) created the first split into “yes” or “no”. The terminal node represents those having viral hepatitis (yes). Among patients without viral hepatitis (no), weight loss, statin, abdominal pain or discomfort, raised ALT, low platelet were the subsequent nodes for the classification decision.
